# Supplementary material for: Developing CRISPR/Cas9-Mediated Fluorescent Reporter Human Pluripotent Stem-Cell Lines for High-Content Screening
Source: Molecules. 2022 Apr 9;27(8):2434. doi: 10.3390/molecules27082434 (PMC9025795; doi:10.3390/molecules27082434)
Supplement: Supplementary file 1 [file molecules-27-02434-s001.zip › molecules-1639120-supplementary.pdf]

molecules

MDPI

*Supplementary Materials*

# Developing CRISPR/Cas9-Mediated Fluorescent Reporter Human Pluripotent Stem-Cell Lines for High-Content Screening

**Kinga Vojnits [1], Mio Nakanishi [1], Deanna Porras [1], Yeonjoon Kim [1], Zhuohang Feng [1], Diana Golubeva [1] and Mick Bhatia [1,*]**

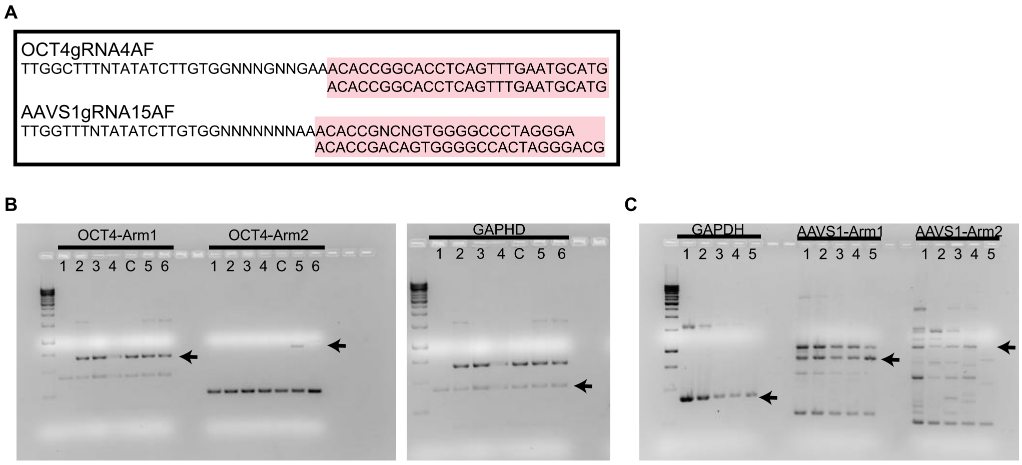


**Figure S1: Generation of EGFP hPSC lines. A)** Sequencing outcomes at the genomic target site. Red highlightes the confirmed matched sequences. **B)** Genotyping PCR for 5′ arm of insertion using primers 5′F and 5′R (740 bp), and 3′ arm of insertion using primers 3′F and 3′R (852 bp) of the selected clones. Two of these clones were correctly targeted at both the 5′ and 3′ ends as determined using PCR genotyping. Similar GAPDH (424 bp) level was determined in all clones. C, indicated parental hPSC. **C)** Genotyping PCR for 5′ arm of insertion using primers 5′F and 5′R (1005 bp), and 3′ arm of insertion using primers 3′F and 3′R (1484 bp) of the selected clones. Similar GAPDH (424 bp) level was determined in all clones. Four of these clones were correctly targeted at both the 5′ and 3′ ends as determined using PCR genotyping. The black arrows indicate the diagnostic band for correct insertion.

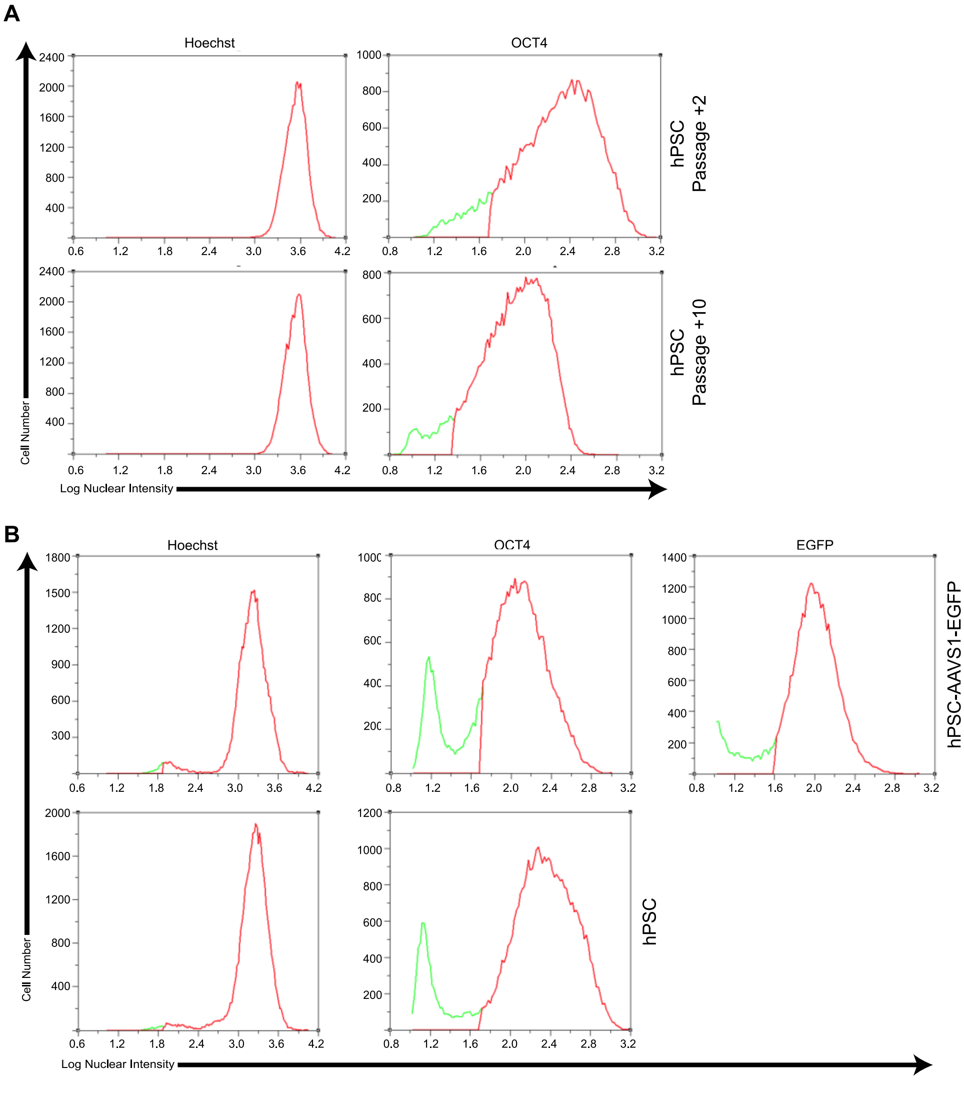


**Figure S2: Generation of EGFP hPSC lines. A)** Typical histograms of the wild-type hPSC show nuclear intensity and frequency of Hoechst+ and OCT4+ cells during culture. **B)** Representative histograms of Hoechst, OCT4, and EGFP of hPSC-AAVS1-EGFP lines and in the wild-type hPSC. Both cell lines exhibited similar OCT4 and nuclear intensity level.

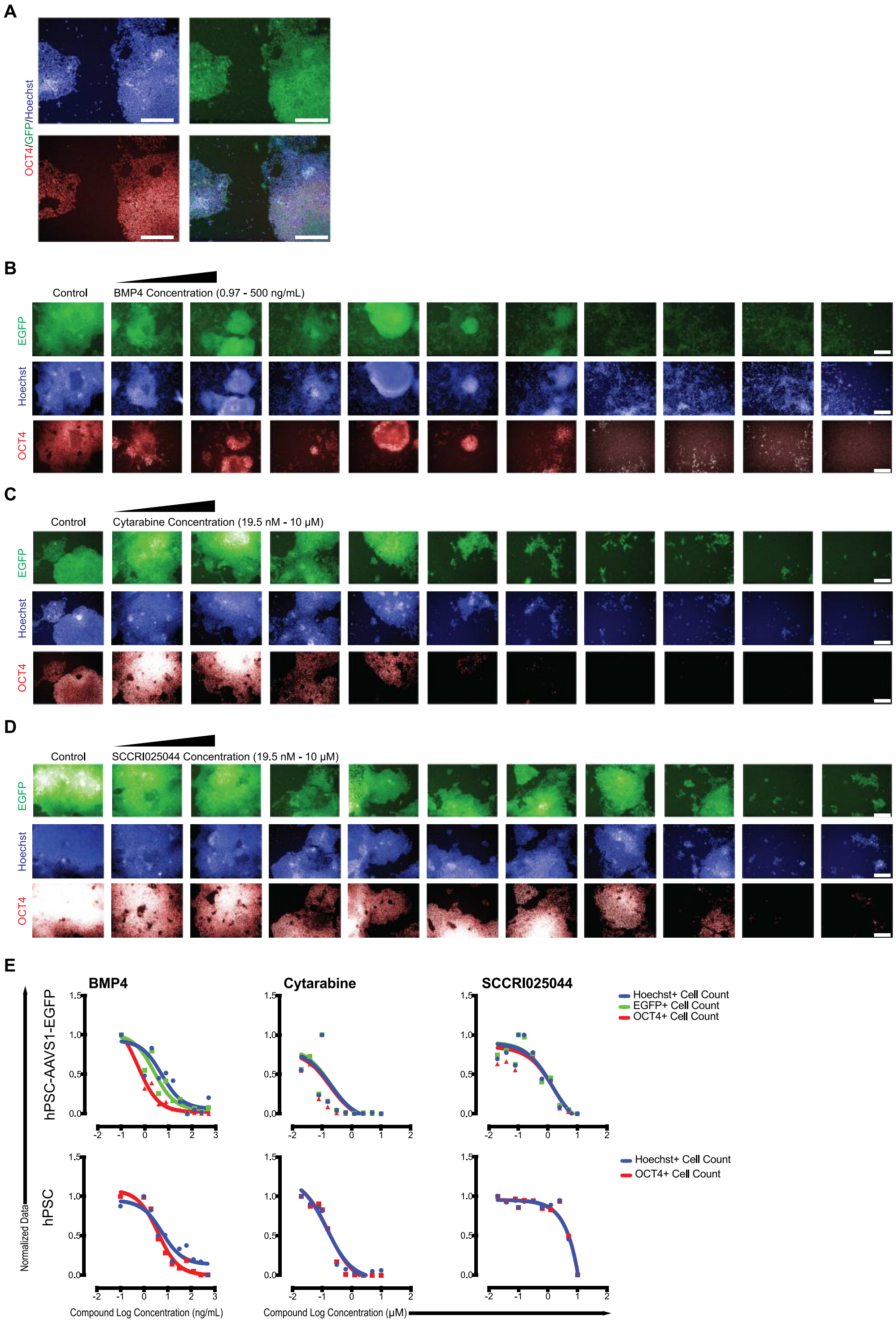


**Figure S3: Adaption of hPSC-AAVS1-EGFP for high-content screening. A)** Representative immunofluorescence images of cells cultured in multi-well formats expressing OCT4 (red) and EGFP (green). Nuclei are stained with Hoechst (blue), scale bars = 80 µM. **B - D)** Representative immunofluorescence images of the responses of hPSC-AAVS1-EGFP line treated with (B) BMP4, (C) Cytarabine, and (D) SCCRI025044. OCT4 (red), EGFP (green), nuclei are stained with Hoechst (blue), scale bars = 80 µM. **E)** Dose-response curves of the responses on hPSC-AAVS1-EGFP versus the parental wild-type hPSC. hPSC-AAVS1-EGFP line shows similar effects and responses to these drug treatments as seen in the parental wild-type hPSC.

| BMP4 | hPSC-AAVS1-EGFP<br>EC50 (ng/mL) | hPSC<br>EC50 (ng/mL) |
|---|---|---|
| Hoechst+ Cell Count | 5.18 | 5.75 |
| OCT4+ Cell Count | 0.51 | 3.87 |
| GFP+ Cell Count | 4.46 | N/A |
| Cytarabine | hPSC-AAVS1-EGFP<br>EC50 (μM) | hPSC<br>EC50 (μM) |
| Hoechst+ Cell Count | 0.2 | 0.14 |
| OCT4+ Cell Count | 0.19 | 0.16 |
| GFP+ Cell Count | 0.18 | N/A |
| SCCRI025044 | hPSC-AAVS1-EGFP<br>EC50 (μM) | hPSC<br>EC50 (μM) |
| Hoechst+ Cell Count | 1.39 | 3.09 |
| OCT4+ Cell Count | 1.31 | 2.81 |
| GFP+ Cell Count | 1.57 | N/A |

**Table S1:** Comparison of the calculated half-maximal effective concentration ($EC_{50}$) of BMP4, Cytarabine and SCCRI025044 tested on hPSC-AAVS1-EGFP and the parental wild-type hPSC line.
